# Polarization spatial diversity and multiplexing MIMO surface enabled by graphene for terahertz communications

**DOI:** 10.1515/nanoph-2025-0204

**Published:** 2025-07-15

**Authors:** Jianzhou Huang, Xudong Wu, Chenjie Xiong, Jia Zhang, Bin Hu

**Affiliations:** School of Optics and Photonics, Beijing Institute of Technology, Beijing 100081, China; National Key Laboratory on Near-Surface Detection, Beijing 100072, China; China Academy of Aerospace Science and Innovation, Beijing 100089, China

**Keywords:** terahertz communication, multiple input multiple output (MIMO), metasurface, graphene

## Abstract

The terahertz (THz) frequency band has abundant spectrum resources, which is suitable for constructing communication systems with ultra-high data rates and extremely low latency. Multiple input multiple output (MIMO) devices are crucial for realizing THz communication, and the synchronous transmission and noncorrelation of different channels are the keys to MIMO technology. This paper proposes a graphene-based polarization spatial diversity and multiplexing MIMO surface (PDM-MIMOS) with 2 × 2 metasurface arrays. Dual-polarized channels can be modulated synchronously by the same metasurface modulator and received by the receiver (RX) without crosstalk. Experimental results demonstrate that the modulation cut-off frequency can reach up to 30 kHz. By constructing a continuous THz wave communication system, it is demonstrated that PDM-MIMOS can achieve spatial diversity and multiplexing, thereby improving communication quality and data rate. Furthermore, we compare the signal quality of THz communication and visible light communication under villainous weather conditions. The experiment proves that the communication reliability of THz communication is 19.4 times that of visible light communication. This work offers potential for compact, dual-polarized modulators that can be applied in THz communication, detection, and imaging.

## 1 Introduction

Recently, there has been a growing demand for high-quality and high-speed transmission in wireless communication [1], [2]. The THz frequency band has received significant attention in both industry and academia due to its potential to build communication systems that meet ultra-high data rates and extremely low latency requirements [3], [4], [5]. THz waves typically refer to electromagnetic waves with frequencies ranging between 0.1 THz and 10 THz. Utilizing the THz band for communication can effectively alleviate the increasingly strained spectrum resources and current capacity limitations of wireless systems, potentially overcoming existing bandwidth bottlenecks to meet the demands of wireless communication [2], [6], [7], [8]. However, due to their shorter wavelengths and weaker diffraction capabilities compared with millimeter waves, THz waves are less capable of penetrating or bypassing obstacles, making traditional communication terminal designs not directly applicable to THz communication.

To address these challenges, researchers have proposed multiple input multiple output (MIMO) technology [9], [10], [11]. The advantages of MIMO technology include information-directional transmission, increased communication capacity [11], [12], and enhanced communication quality [13]. However, recent MIMO devices are mainly focused on the needs of millimeter waves, and the reconfigurable intelligent surfaces designed with PIN diodes have a wide fabrication linewidth, which makes it difficult to meet the processing accuracy of THz devices.

Compared to 5G communication, terminal devices in the THz band require smaller geometric dimensions and higher portability. Thus, metasurfaces offer a new approach to solve these issues. Metasurfaces consist of subwavelength antenna arrays made of metals or dielectrics, forming a two-dimensional artificial structure [14], [15], [16], [17]. They possess advantages of small size, light weight, high integration, and flexible control of electromagnetic waves, facilitating the realization of highly integrated THz communication devices. However, tunability of metasurfaces is still highly demanded for THz communication [18].

To realize configurable THz metasurfaces, researchers have adopted active materials, including electric tuning using liquid crystals [19], [20], [21], diodes [22], and 2D materials [23]; thermal tuning using phase change materials [24], [25]; and optical tuning using perovskites [26]. Graphene, as a rapidly developing 2D material, offers advantages including high mechanical strength, ease of fabrication, fast modulation up to 427 GHz [27], and low absorption in the THz frequency range, enabling the possibility of low-cost and lightweight THz communication devices with high speed [28], [29], [30], [31]. Various concepts of MIMO communication devices based on graphene have been proposed in recent years, which typically consist of active graphene elements placed over a metallic ground layer, with a dielectric layer in between [32], [33], [34], [35]. However, it is not easy to implement these devices in practice because applying vertical electric fields to an element array with independent graphene gating for each element is challenging [30]. Besides, using ionic gels to apply the electric fields also suffers from extremely low modulation speeds (<1 Hz) [36]. Therefore, it is essential to explore effective strategies for implementing graphene-based MIMO communication in the THz band.

Additionally, while MIMO communication achieves higher data rates and better communication quality, it increases the complexity of signal processing at the RX due to the need of solving interference from simultaneous signals. Therefore, a certain distance (>*λ*/2) needs to be satisfied between different MIMO units. This presents a challenge to the integration of MIMO devices. To address this issue, researchers proposed the polarization diversity and multiplexing MIMO system utilizing mutually orthogonal polarized electromagnetic waves [37], [38]. Moreover, stringent synchronization requirements between two modulated signals are necessary to leverage the advantages of space-time coding and multiuser MIMO transmission [39].

**Figure 1:**
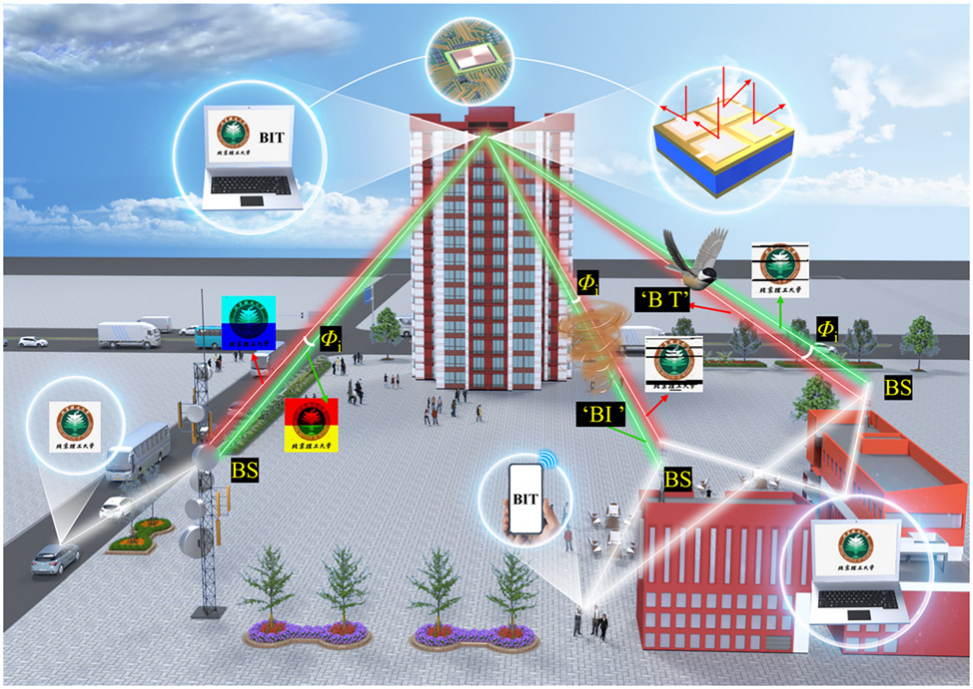
Conceptual scheme of the PDM-MIMOS in practical applications. The red and green lines are transmission channels with different polarizations, respectively. BS denotes Base Station.

To solve these problems, this study proposes a highly integrated graphene-based polarization diversity and multiplexing MIMO surface (PDM-MIMOS), which ensures that the RX receives two polarization diversity channels without correlation and realizes the complete synchronous transmission of information. Figure 1 illustrates the concept that due to the noncorrelation of different polarization channels, signals of different polarization directions received by the same RX do not interfere with each other. Additionally, the PDM-MIMOS utilizes spatial diversity to reduce interference during communication, thereby enhancing the communication quality. Moreover, through spatial multiplexing, the same information can be transmitted through multiple channels simultaneously, increasing the communication speed. Our designed 2 × 2 PDM-MIMOS achieves 8-channel polarization spatial diversity and multiplexing. By modulating graphene, we can achieve THz communication with a maximum cutoff frequency of 30 kHz. In the end, we compared the communication quality of THz and visible light in villainous weather conditions, revealing that the signal reliability of THz communication is 19.4 times higher than that of visible light communication. We hope this work presents a viable design and experimental approach for THz MIMO communication based on graphene, along with feasible optimization strategies.

## 2 Unit cell design and simulation

Leveraging polarization diversity and multiplexing presents an innovative method to enhance the transmission capacity of THz communication. By exploiting these techniques, potential interference issues can be effectively mitigated, leading to more reliable and efficient communication in THz systems. Meanwhile, signals of different polarizations detected by the same RX do not interfere with each other. Therefore, the distance between MIMO receivers can be significantly reduced. To achieve multichannel transmission, it is necessary to divide the parallel-polarized and cross-polarized THz waves into two directions. Therefore, in this work, we elaborately design the metasurface to realize the specular reflection of the parallel-polarized THz wave and the anomalous reflection of the cross-polarized THz wave.

**Figure 2:**
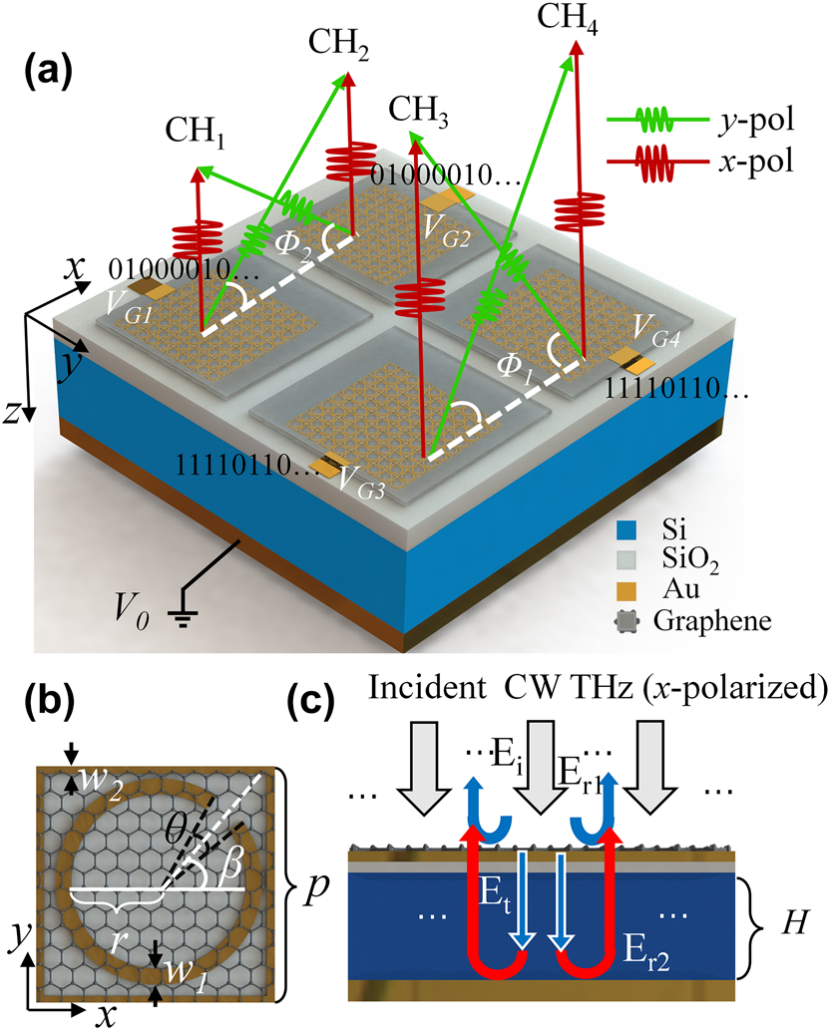
Schematic of the PDM-MIMOS. (a) Proposed reflective MIMO modulator for THz communication. The metasurface arrays of CH_1_ (CH_3_) and CH_2_ (CH_4_) are mirror-symmetric about the *y*-axis. (b) Top view of one unit cell with the C-shaped and rectangular antenna. The thickness of SiO_2_ and gold is 30 nm and 100 nm, respectively. (c) Side view of the structure. *H* = 240 μm.

Figure 2a demonstrates the schematic of the proposed reflective PDM-MIMOS. The structure consists of 2 × 2 metasurfaces (CH_1_, CH_2_, CH_3_, and CH_4_) covered with four electrically isolated graphene. Each metasurface enables two communication channels with polarization directions that are mutually perpendicular to each other. The incident wave is *x*-polarized. The red arrows represent specular reflected parallel-polarized THz waves (*x*-polarized), while the green arrows represent anomalously reflected cross-polarized THz waves (*y*-polarized).

The metasurface is fabricated on a slightly doped N-type silicon substrate (1–10 Ω cm) with thickness *H* = 240 μm, and the permittivity is measured as *ε*_1_ = 11.1 + 0.836*i* using a THz time-domain spectrometer (CIP-TDS, Daheng New Epoch Technology, Inc.). The thickness of the dielectric layer SiO_2_ is 30 nm, and the permittivity is measured as *ε*_2_ = 3.80 + 0.051*i*. The bottom of the Si substrate is covered with a gold layer as a reflector. As shown in Figure 2b, each unit cell consists of a C-shaped gold antenna in a gold rectangle covered by graphene. The C-shaped antenna can convert part of the incident *x*-polarized THz wave into *y*-polarization of the reflected wave with an additional phase dependent on the opening angle *θ*, while the reflected *x*-polarization has no phase modulation [17]. The width of the C-shaped antenna is *w*_1_ = 10 μm, and that of the rectangle is *w*_2_ = 5 μm. The opening direction is *β* = ±45°, the radius is *r* = 55 μm, and the period is *p* = 150 μm. The dimension of the PDM-MIMOS is 23.6 mm × 23.6 mm, the distance between the two adjacent metasurfaces is *d* = 2 mm, and the dimension of every metasurface array is 10.8 mm × 10.8 mm, which consists of 72 × 72 unit cells.

To load the digital signals onto an incident continuous-wave (CW), it is necessary to modulate the intensity of the reflected *x*- and *y*-polarizations. Therefore, a vertical static electric field is applied on graphene to modify its electrochemical potential and the interaction between THz waves and the unit cells [30]. In Figure 2a, the reflective layer is grounded (*V*_0_), and the Raspberry Pi applies the output electrical signal (*V*_G1_–*V*_G4_) after digital-to-analog conversion (DAC) to the graphene, thus loading the digital signal on the reflected wave to achieve THz communication.

To achieve polarization diversity and multiplexing, enhancing the conversion of cross-polarized THz waves is the key issue. Therefore, we introduce a Fabry–Pérot (F–P) cavity to reinforce the interaction between THz waves and the metallic metasurface, as shown in Figure 2c. After the linearly *x*-polarized THz wave shines on the metasurface, part of the THz wave is reflected, and the transmitted wave (*E*_*t*_) is reflected by the bottom gold layer to interact with the metasurface again. Therefore, more cross-polarized (*y*-polarized) THz waves are excited to achieve enhanced efficiency [40], [41].

**Figure 3:**
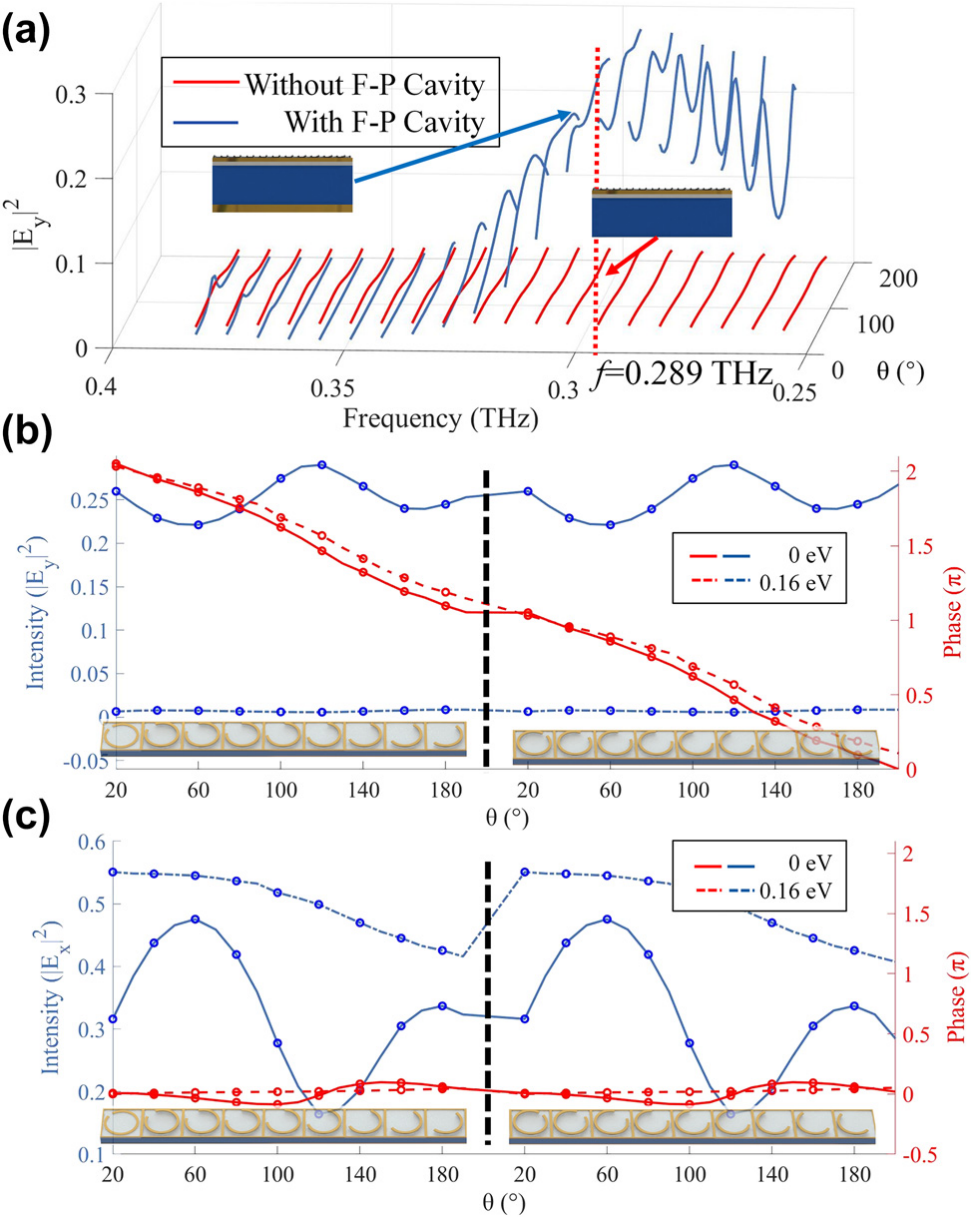
Simulation results of the unit cells. (a) Simulated *y*-polarization reflectance spectra of unit cells at different opening angles *θ* with and without a F–P cavity. The incident wave is *x*-polarized. (b)–(c) Intensity and phase shift of *E*_*x*_ (copolarization) and *E*_*y*_ (cross-polarization) at different opening angles *θ* with graphene chemical potentials of *E*_F_ = 0 eV and *E*_F_ = 0.16 eV.

Figure 3a demonstrates that the F–P cavity significantly improves the conversion efficiency and enhances cross-polarization intensity. The simulation results were conducted by the Lumerical FDTD Solutions software. The boundary conditions are set to periodic in the *x* and *y* directions and perfectly matched layers (PML) in the *z* direction. The mesh of the C-shaped antenna and graphene is set to 1 μm, 1 μm, and 0.2 μm in the *x*, *y*, and *z* directions, respectively, and the auto-shutoff is set to 10^−5^.

The solid red lines and blue lines indicate the reflectance spectra of unit cells at different opening angles *θ* without and with the F–P cavity. At 0.289 THz, the intensity of *y*-polarization is less than 0.02 without the F–P cavity at all opening angles. In contrast, the *y*-polarization intensity with the F–P cavity is higher than 0.22. It is evident that the intensity |*E*_*y*_|^2^ increases 11–21 times at frequency *f* = 0.289 THz with different opening angles. In addition, we also calculated the absorption of different unit structures, as shown in Figure S1 (see Supporting Information). The main energy loss of PDM-MIMOS is due to the absorption of THz waves by slightly doped silicon. The absorption of different unit structures ranges from 30 % to 60 %.

The PDM-MIMOS implementation must meet two conditions: binary intensity modulation of the *x*-polarized and *y*-polarized THz signals, and the phase gradient design of specular and anomalous reflection. Next, we simulated the reflection spectra with different C-shaped antenna opening angles at the frequency of *f* = 0.289 THz, as shown in Figure 3b and c. The solid lines denote the simulation results of the unit cells under the graphene chemical potential of 0 eV, while the dashed lines correspond to the chemical potential of 0.16 eV. When the graphene chemical potential changes from 0 eV to 0.16 eV, the phase shift of both *y*-polarized and *x*-polarized THz waves is insignificant. However, the intensity of the *y*-polarized THz waves decreases dramatically with the increase of graphene chemical potential, while the intensity of the *x*-polarized THz waves increases, demonstrating the polarization conversion of the unit cells is decreased with doped graphene. This satisfies the condition of binary intensity manipulation. Moreover, the phase of THz waves reflected by unit cells with different opening angles barely changes for the *x*-polarization but covers a 2*π* phase variation in the *y*-polarization. As a result, it is possible to design the phase gradient of anomalous reflection for cross-polarization. It is worth mentioning that the phase shift of the reflected cross-polarization changes by *π* when the opening direction is rotated 90° along its central axis, without any variation in the reflection amplitude [17].

## 3 MIMO device design and simulation

Dual channel reflection of *x*-polarized and *y*-polarized waves is the key to achieving polarization spatial diversity and multiplexing in THz communication, as shown in Figure 2a. Therefore, we design periodic superlattices with different phase gradients based on the phase shift results of the cross polarization in Figure 3. According to the generalized Snell’s law [42], the deflection direction can be manipulated by designing the phase gradient.

(1) $$\sin\Phi_{r}-\sin\Phi_{i}=\frac{\lambda }{2\pi n_{i}}\frac{\text{d}\varphi }{dx}$$

where *λ* is the wavelength of the incident wave, Φ_*r*_ is the reflection angle, Φ_*i*_ is the incident angle, and *n*_*i*_ is the refractive index of the incident/reflection medium. d*φ*/d*x* is the phase gradient along the *x*-direction. When an *x*-polarized THz wave is incident, there will be no phase modulation for the *x*-polarized reflective wave. Thus, the phase gradient approaches 0, and Φ_*r*_ = Φ_*i*_. For the *y*-polarized reflective wave, the equation can be further simplified if the THz wave is normally incident from air:

(2) $$\Phi_{r}=\arcsin\left(\frac{\lambda }{Np}\right)$$

where *p* is the period of the unit cells. *N* is the number of the unit cells selected for 2*π* phase variation. In this work, *N* = 8 and *N* = 18 are selected to form two different superlattices, as shown in Table S1 (see Supporting Information). According to the above equation, the abnormal reflection angles are Φ_*r*_ = 60° (*N* = 8) and 22.6° (*N* = 18). In our design, CH_1_ and CH_2_ are composed of Superlattice 1, while CH_3_ and CH_4_ are composed of Superlattice 2.

**Figure 4:**
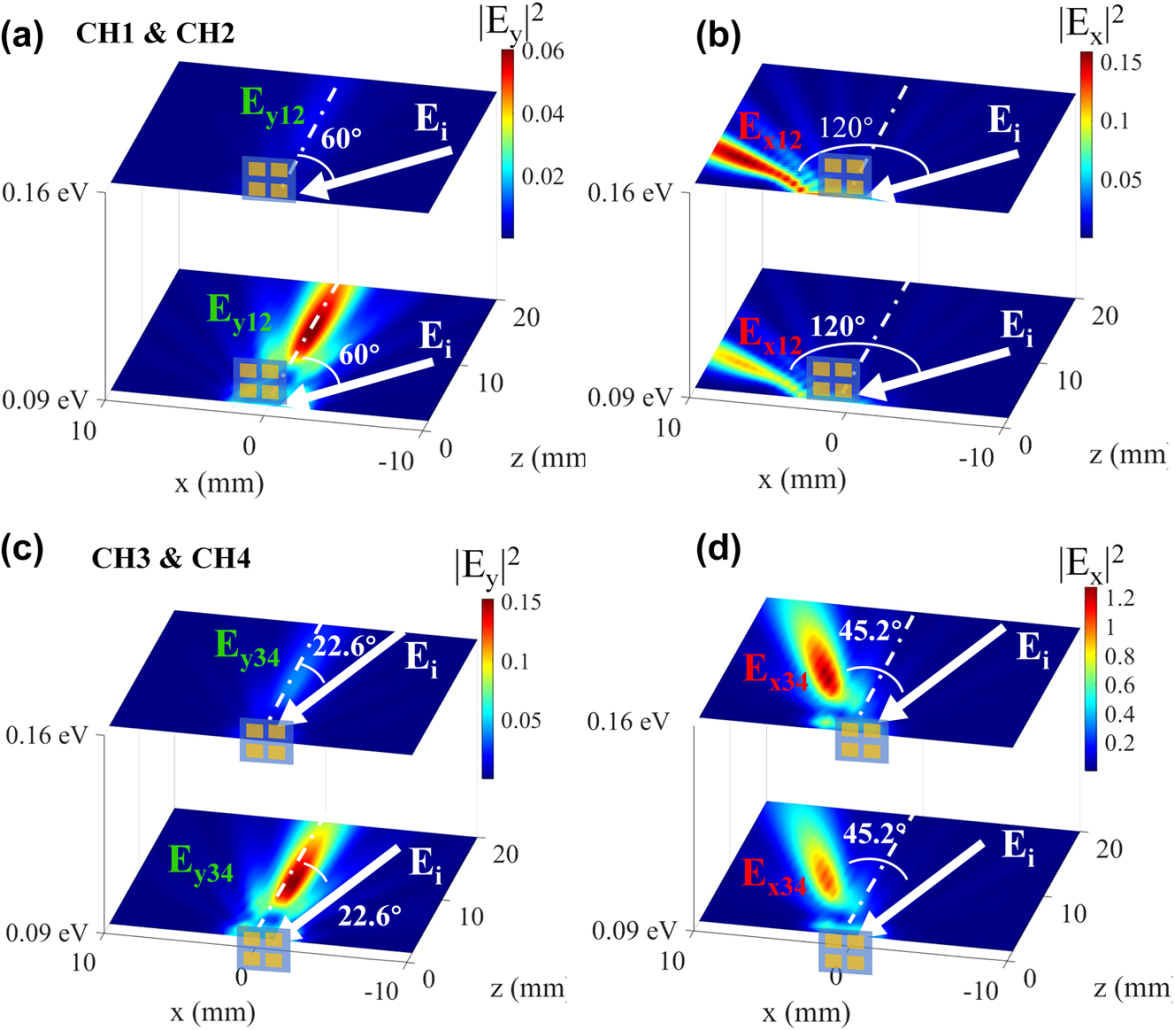
Simulated PDM-MIMO function of the proposed device. (a)–(d) Simulation results of reflected intensity distributions in the *y*–*z* plane with graphene chemical potential of *E*_F_ = 0 eV and *E*_F_ = 0.16 eV. The incidence angles of CH_1_ (CH_2_) and CH_3_ (CH_4_) are 60° and 22.6°, respectively. The red circle highlights the device region illuminated by the THz wave.

We verified the tunability of our designed 2 × 2 modulator by conducting far-field simulations. The incident light was an *x*-polarized wave with different incident angles at the frequency *f* = 0.289 THz, marked by white arrows. The metasurface arrays of CH_1_ and CH_2_ (CH_3_ and CH_4_) are symmetric about the *y*-axis, so the phase gradient and the far-field intensity distribution are also symmetric about the *y*-axis. Therefore, we only demonstrate the results of CH_1_ and CH_3_ in Figure 4. Abnormal and specular reflections can be observed under different chemical potentials. When the incident wave illuminates on CH_1_ (CH_2_) and CH_3_ (CH_4_) at the incidence angles of 60° and 22.6°, respectively, it can be seen that *E*_*y*_ has abnormal reflection (reflection angle equals 0°) and *E*_*x*_ has specular reflection (reflection angle equals incident angle). Additionally, by adjusting the chemical potential (*E*_F_) within the range of 0.09 eV–0.16 eV, it becomes evident that the abnormal reflected intensity of the *y*-polarization experiences a significant reduction. In contrast, the intensity of the *x*-polarized specular reflected wave is increased. This opposing modulation effect on reflective *y*-polarization and *x*-polarization can be attributed to the alteration in the graphene chemical potential, which in turn changes the conversion rate of cross-polarized THz waves.

Therefore, intensity modulation and reflection separation of the 2 orthogonal polarizations can be achieved simultaneously through the same metasurface array. This means that the same modulator can achieve the modulation of dual-polarized channels without crosstalk. The results demonstrate the feasibility of simultaneously modulating dual polarization channels.

**Figure 5:**
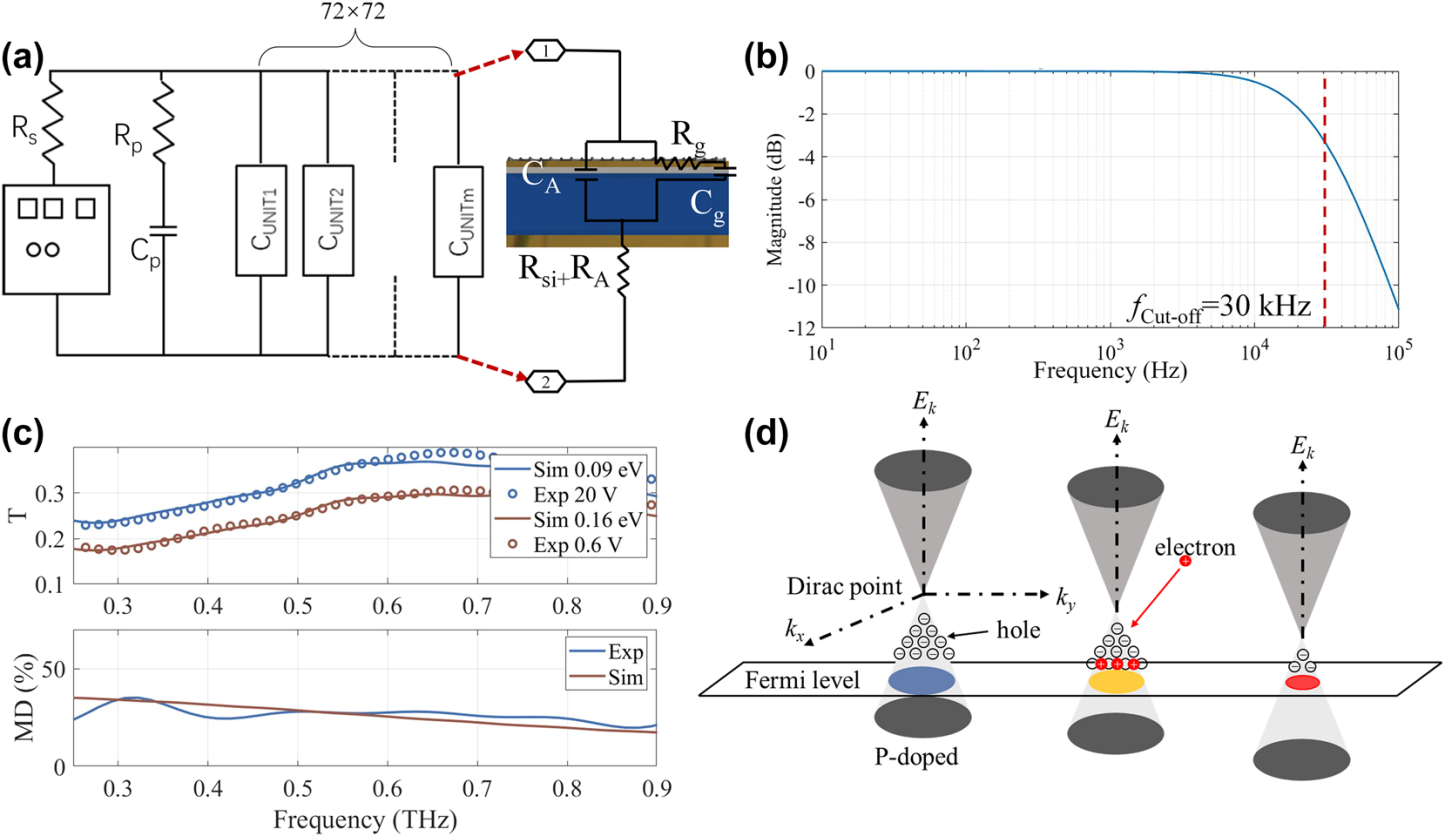
Modulation performance of the proposed device. (a) Lumped element circuit model of the MIMO device. *C*_p_ = 50 pF, *C*_g_ = 25 pF, *R*_p_ = 100 Ω, *R*_g_ = 500 Ω, *C*_A_ = 5 fF, *R*_A_ + *R*_Si_ = 133 Ω. (b) Transfer function of the MIMO device. *f*_cut-off_ = 30 kHz. (c) Experimental and simulated results of transmission spectra and modulation depth (MD) modulated by monolayer graphene on N-type silicon substrate. (d) The energy band structure and Fermi plane near the Dirac point move with a positive gate voltage applied.

To achieve wireless communication, the bandwidth of the modulator is also very important. Figure 5a and b demonstrates the lumped element circuit model and the speed characteristics of the PDM-MIMOS device. The output impedance of the AC voltage is *R*_s_ = 50 Ω. The device is modeled as 72 × 72 parallel elements, where *C*_p_ is the capacitance of the electronic gate voltage pads. *R*_p_ is the gate pad resistance to each of these pads. The inset of Figure 5a shows one of the subcircuits. *R*_si_ denotes the unit cell resistance through the N-type silicon, calculated using Pouillet’s law. *R*_A_ is the back pad resistance. *C*_A_ and *C*_g_ represent the gold antenna pad and graphene capacitances, respectively.

The capacitance *C*_g_ of each unit cell is given by the standard formula for the parallel plate capacitor *C*_g_ = *ε*_0_ *ε*_*r*_·*S*/*d*, where *ε*_0_ is the vacuum permittivity, *ε*_*r*_ = 3.8 is the relative dielectric constant of SiO_2_, *S* is the total graphene area, and *d* is the thickness of the SiO_2_ dielectric layer. Meanwhile, *R*_g_ is the average graphene resistance for each unit cell. We applied SIMULINK to solve the circuit characteristics, as shown in Figure 5b. The cut-off frequency is the −3 dB value of the voltage transferred across the graphene capacitors from the total circuit [43]. The simulation results illustrate that the cut-off frequency of our device is *f*_cut-off_ = 30 kHz. Although this is a simplified circuit model, it provides an approximate cut-off frequency for THz communication. On the other hand, it provides a way to optimize the design and improve the cutoff frequency. For example, the cut-off frequency can be increased by increasing the thickness of the SiO_2_ layer and reducing *C*_g_ through patterning the graphene.

Before the communication test of the PDM-MIMOS, the correspondence between the graphene chemical potential and the applied external voltage needs to be verified. Thus, we transferred monolayer graphene onto an N-type silicon substrate and modulated it by applying a vertical electric field. The measurement is performed by the CIP-TDS as demonstrated in video 1 (see Supporting Information).

Figure 5c presents the transmission spectra and modulation depth (MD) from both simulation and experiment. These demonstrate that the applied voltages of 20 V and 0.6 V correspond to chemical potentials of 0.09 eV and 0.16 eV, respectively. As the applied gate voltage increases, graphene’s Fermi level shifts toward the Dirac point (it is worth noting that the chemical potentials of graphene are all defined as absolute values). This phenomenon is attributed to the atmospheric adsorption and the connection between the electrode and graphene using a silver paste, which has a high electron affinity, resulting in p-type doping [44], [45]. Since the back-gate connects to the DC source’s positive terminal and the graphene electrode to the negative terminal, the number of holes decreases and graphene’s Fermi level shifts toward the Dirac point, increasing THz transmission as shown in Figure 5c and d. The experimental and simulation modulation depths are defined as |*T*_20V_–*T*_0.6V_|/*T*_20V_ and |*T*_0.09eV_–*T*_0.16eV_|/*T*_0.09eV_, respectively. The results show that the modulation depth of simulation agrees with that of the experiment in the wide band over 0.6 THz, demonstrating the correspondence between the graphene chemical potential and gate voltage.

## 4 Fabrication and measurement

### 4.1 Device fabrication

**Figure 6:**
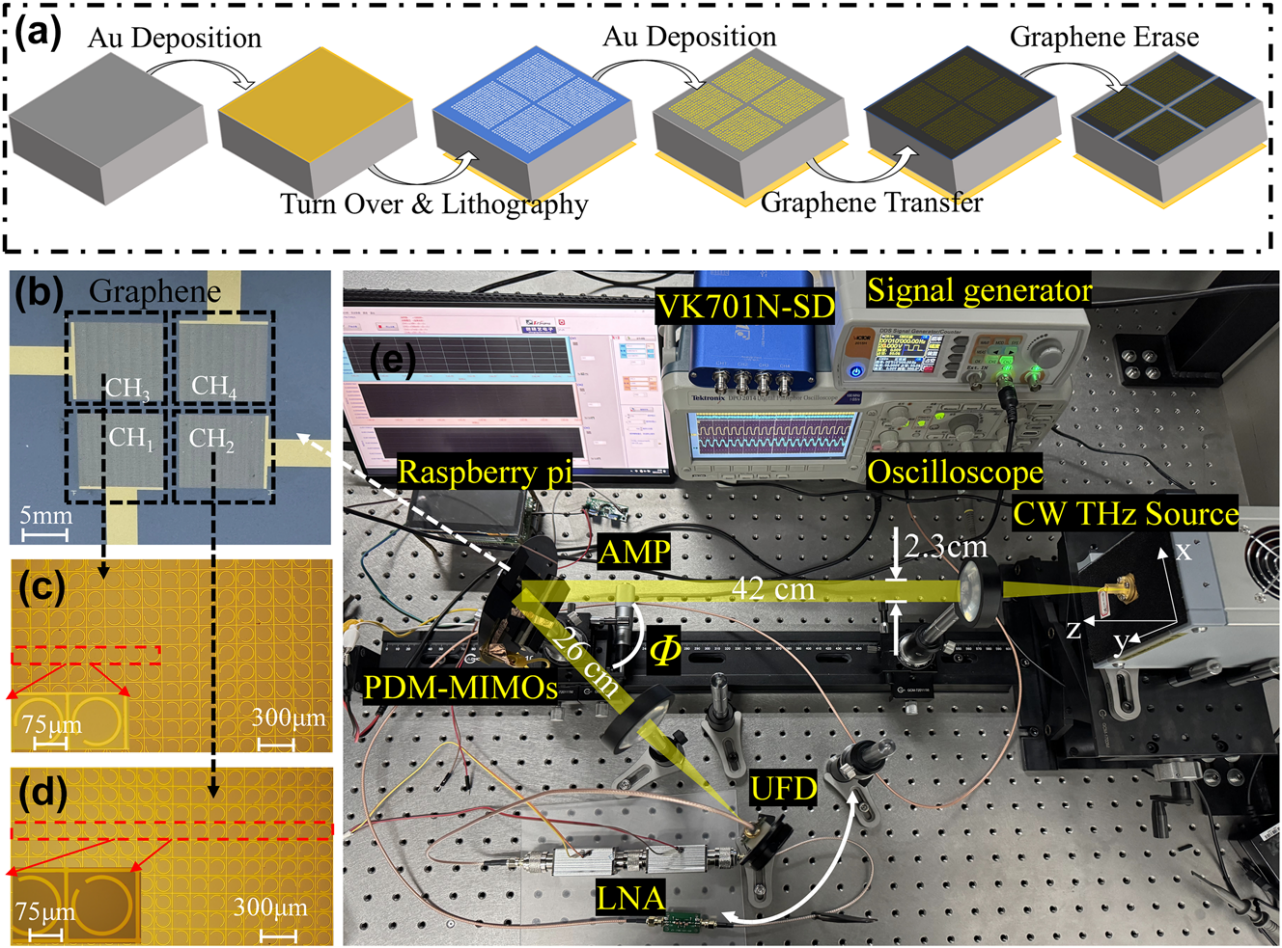
Fabrication and measurement setup. (a) Fabrication process of the MIMO device. (b) Fabricated four-channel MIMO device. (c) and (d) Optical images of CH_3_ and CH_2_ under a microscope. (e) CW THz communication system. The frequency of the CW THz source is *f* = 0.289 THz.

Figure 6a shows the fabrication process of the designed PDM-MIMOS. We first prepared a N-type silicon substrate with a thickness of 240 μm. A layer of S1813 photoresist was then spin-coated on the silicon wafer at a speed of 500 r/min for 10 s, followed by 3,000 r/min for 60 s. Then, it was dried at 115 °C for 60 s. Second, a UV lithography system produced the pattern on the photoresist under a Cr-plated quartz mask. In the third step, we deposited a 10 nm Cr layer and a 90 nm gold layer onto the photoresist by electron beam evaporation coating technology. The Cr layer acted as an adhesive. The vacuum pressure was 5 × 10^−6^ Torr, and the deposition rate was 0.5 Å/s (1 Å = 0.1 nm). Finally, the gold array was obtained by passing a lift-off process. The monolayer graphene, synthesized by chemical vapor deposition (CVD) technology, was transferred to the fabricated metasurfaces and divided into four areas by a cotton swab soaked with alcohol to realize electrical isolation. The electrical isolation between the four graphene patches is demonstrated in Figure S2 (see Supporting Information). Figure 6b is the photograph of the fabricated PDM-MIMOS. Figure 6c and d shows the photographs of the antenna array after fabrication under a metalloscope (MGL6000). The red dashed boxes indicate superlattice 1 with 8 unit cells and superlattice 2 with 18 unit cells.

The CH_1_ and CH_2_ arrays were designed symmetrically to accommodate multiple inputs. For instance, the *y*-polarized signal modulated by CH_1_ and the *x*-polarized signal modulated by CH_2_ can be received by the same RX, as shown in Figure 2a. The electrical signals after DAC were applied to the electrically isolated graphene, indicated by black dashed boxes in Figure 6a, using four rectangular metal electrodes.

The THz communication testing system is shown in Figure 6e. The polarization direction of the outgoing THz wave is along the *x*-axis, the propagation direction is along the *z*-axis, and the polarization direction of the cross-polarized THz wave excited by PDM-MIMOS is along the *y*-axis. Initially, a Raspberry Pi was employed for the DAC of communication data. Next, an amplifier was used to boost the electrical signal to specific levels (20 V and 0.6 V), which was then applied to the graphene of PDM-MIMOS to achieve binary intensity modulation in both the *x*- and *y*-polarizations simultaneously. This process allowed the PDM-MIMOS to encode communication information onto THz waves emitted by a CW THz source (IMPATT-289-50mW). Subsequently, the modulated wave was detected by a linearly polarized ultrafast detector (UFD, TeraFAST) with a sensitivity of 0.5 V/W, which effectively converted the optical signal into an electrical signal. Finally, the electrical signals were processed using a data acquisition card (VK701N-SD) with a 100 kilo samples per second (KSPS) sampling rate. The processed digital signals were then transmitted to a computer for further data recovery and analysis.

### 4.2 Device characterization

**Figure 7:**
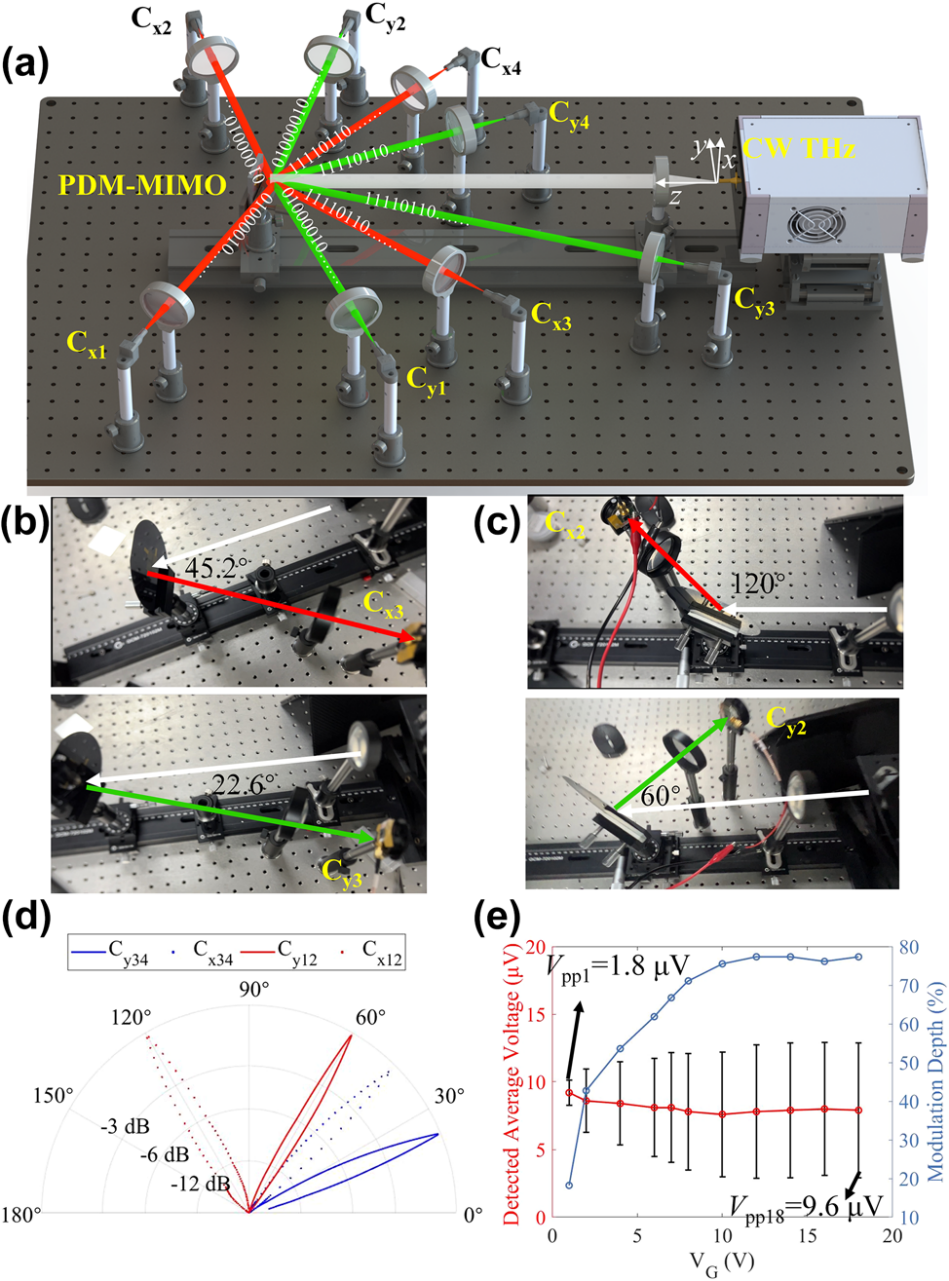
PDM-MIMO performance measurement of the device. (a) Schematic of the measurement for the MIMO device by CW THz communication system. The incidence angle of the CW THz wave is changed by rotating the MIMO device, where the incidence angle of CH_1_ and CH_2_ (CH_3_ and CH_4_) is ±60° (±22.6°). (b) Experimental photographs of C_x3_ channel and C_y3_ channel. (c) Experimental photographs of C_x2_ channel and C_y2_ channel. (d) Measured radiation patterns of the MIMO. (e) The detected average voltage, amplitude (*V*_pp_), and modulation depth of the *y*-polarization signal under different peak-to-peak gate voltages.

To avoid the reflected wave entering the laser source and to facilitate the measurement of specular and abnormally reflected THz signals, we adjusted the incident angle to the designed reflection angle. Thus, we performed modulation measurements on different channels by rotating the device along the *x*-axis so that the incidence angles can be switched to ±60° and ±22.6°, as depicted in Figure 7a. To describe the experimental results more clearly, we used C_xi_ and C_yi_ to represent the different communication channels. For example, the channel represented by C_x3_ and C_y3_ is the *x*-polarized and *y*-polarized THz wave modulated through the CH_3_ with the reflection angle of 45.2° and 22.6° (corresponding to Figure 4c and d), respectively, as shown in Figure 7b. At the same time, C_x2_ and C_y2_ are the *x*-polarized THz and *y*-polarized THz wave modulated through the CH_2_ with the reflection angle of 120° and 60° (corresponding to Figure 4a and b), as shown in Figure 7c. Figure 7d shows the measured reflection angles. The red dashed and solid lines denote the measurement results of C_x12_ and C_y12_ channels at a rotating angle of 60°, which are 120° and 60°, respectively. The blue dashed and solid lines are the measurement results of C_x34_ and C_y34_ channels at a rotating angle of 22.6°, which are 45.2° and 22.6°, respectively.

Therefore, the experimental results are consistent with the design and simulation results, and the deviation of the angular beam pointing of the proposed PDM-MIMOS can be neglected. We also illustrate the misalignment sensitivity based on the data detected from different channels, and the results are shown in Figure S3. As the propagation direction deviates from the nominal propagation axis, the detected energy decreases significantly. When the deviation exceeds 10°, all channels are reduced to −10 dB. As a result, THz MIMO transceivers must be simultaneously pointed at each other for communication to take place, in which precise alignment should be maintained [2].

We applied a 100 Hz sinusoidal signal on CH_3_ through the signal generator (VICTOR 2015H) and changed the peak-to-peak gate voltage to measure the modulation depth of the THz signal. The results of channel C_y3_ are shown in Figure 7e. The red line denotes the detected average voltage by the UFD, and the black lines are the detected peak-to-peak voltages. The blue line denotes the modulation depth calculated by *V*_pp_/(*V*_Average Voltage_ + 0.5*V*_pp_). The results show that the detected peak-to-peak voltage and modulation depth are significantly increased versus the applied gate voltage from 1 V to 12 V, and the modulation depth tends to be saturated with the gate voltage. The highest detected peak-to-peak voltage is 9.6 μV, while the lowest is 1.8 μV. Based on these results, we chose a 15 V peak-to-peak gate voltage for gating graphene to reach the saturated modulation depth.

**Figure 8:**
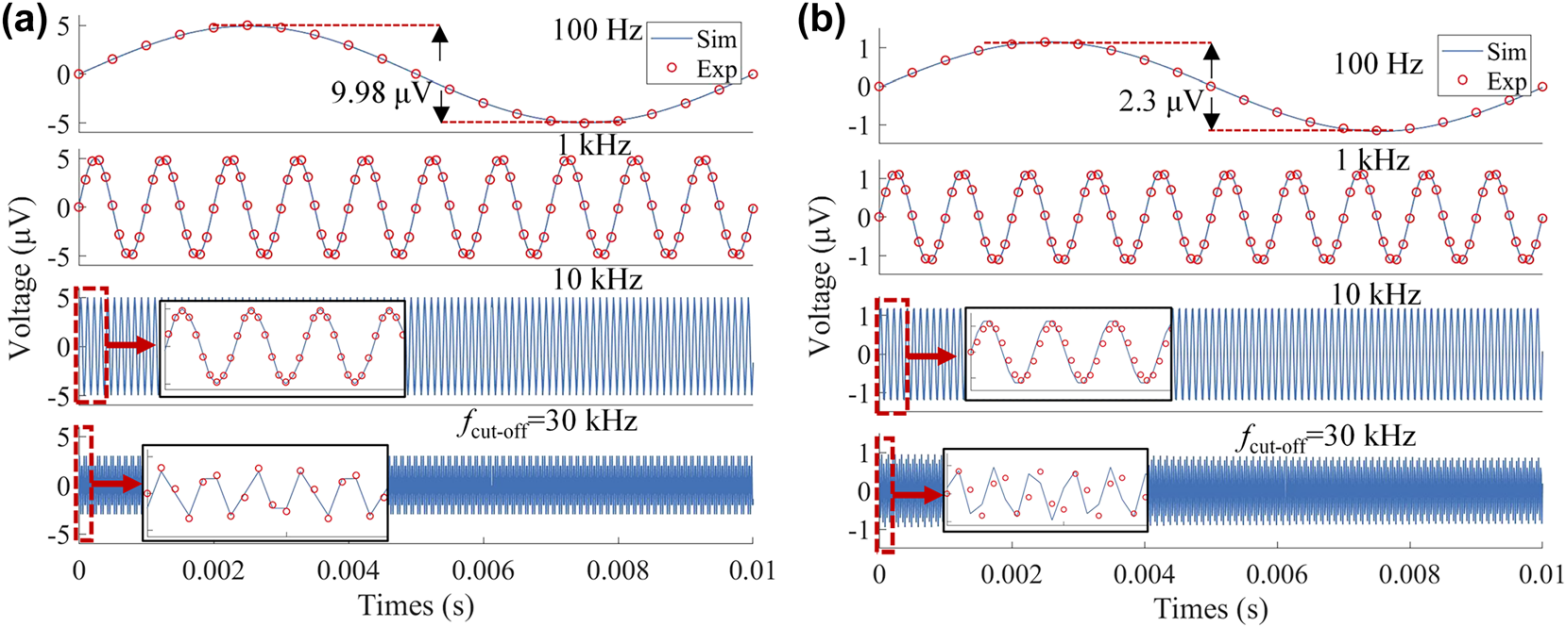
Experimentally detected and circuit-simulated THz signals at different modulation speeds. The peak-to-peak gate voltage is *V*_G_ = 15 V. (a) Gate voltage applied on CH_3_, (b) gate voltage applied on CH_2_.

The experiments were performed in channels C_y3_ (Figure 8a) and C_y2_ (Figure 8b), and the simulation was calculated by SIMULINK. We normalized the simulated sinusoidal peak-to-peak value to the measured signal to facilitate comparison. It is found that the experiment results align with the simulation at modulation frequencies of 100 Hz, 1 kHz, and 10 kHz. The detected peak-to-peak values of C_y3_ and C_y2_ decreased from 9.98 μV and 2.3 μV to 6.86 μV and 1.58 μV as the modulation frequency changed from 100 Hz to 30 kHz. As a result, the peak-to-peak value is reduced to 0.687 times (<−3 dB) of the maximum value, indicating that 30 kHz is close to the cut-off frequency. These results demonstrate that this modulator can load up to 30 Kbps data rate on the carrier wave by one of the metasurfaces. Since the four graphene are electrically isolated from each other, a data rate of 120 Kbps can be achieved through spatial multiplexing.

**Figure 9:**
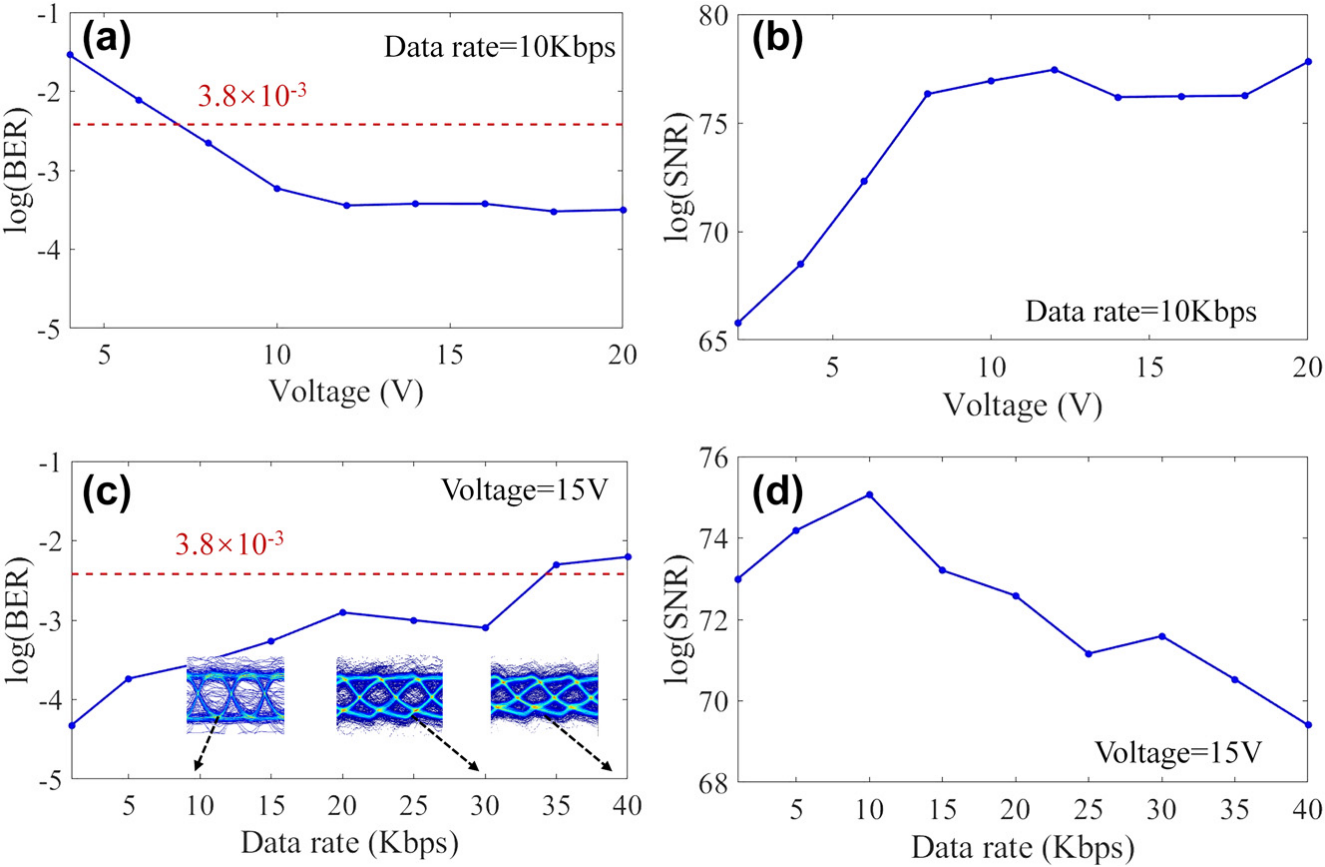
Measurement results of the device modulation. (a)–(b) Measured BER and SNR of CH_1_ metasurface at different data rates. (c)–(d) Measured BER and SNR of CH_1_ metasurface at different signal modulation peak-to-peak values.

Subsequently, we demonstrate the bit error rate (BER) and signal-to-noise ratio (SNR) at different data rates and signal modulation peak-to-peak values to identify the transmission performance of the wireless transmission system in this work, as shown in Figure 9. To amplify the microvolt signal output from the UFD to the millivolt level for detection by the oscilloscope (Tektronix DPO2014), we used a 60 dB low-noise amplifier (LNA), as shown in Figure 6e. The BER is calculated by comparing the received bits after demodulation with the original 211,251 bits.

In Figure 9a and b, we measured the BER and SNR with different peak-to-peak values at a data rate of 10 Kbps. The results show that the BER decreases as the voltage increases, while the SNR improves. These results are consistent with the results in Figure 7e, both indicating that the modulation of graphene with gate voltage reaches saturation at 12 V. Then we measured the BER and SNR at different data rates by applying a 15 V peak-to-peak modulation signal. The BER performance after wireless delivery is less than 3.8 × 10^−3^ when the data rate is less than 35 Kbps, as shown in Figure 9c. The insets in Figure 9c show the measured eye diagrams. It can be seen that the eye diagram is closed after the data rate is higher than 30 Kbps. Meanwhile, as shown in Figure 9d, the SNR decreases as the data rate increases, but it remains above 68 dB, indicating good transmission quality. The main reason for the low data rate is the large graphene area, which results in a large graphene capacitance and subsequently reduces the cut-off frequency of the device. The data rate can be effectively improved using patterned graphene pieces [46].

### 4.3 Performance of polarization spatial diversity and multiplexing

**Figure 10:**
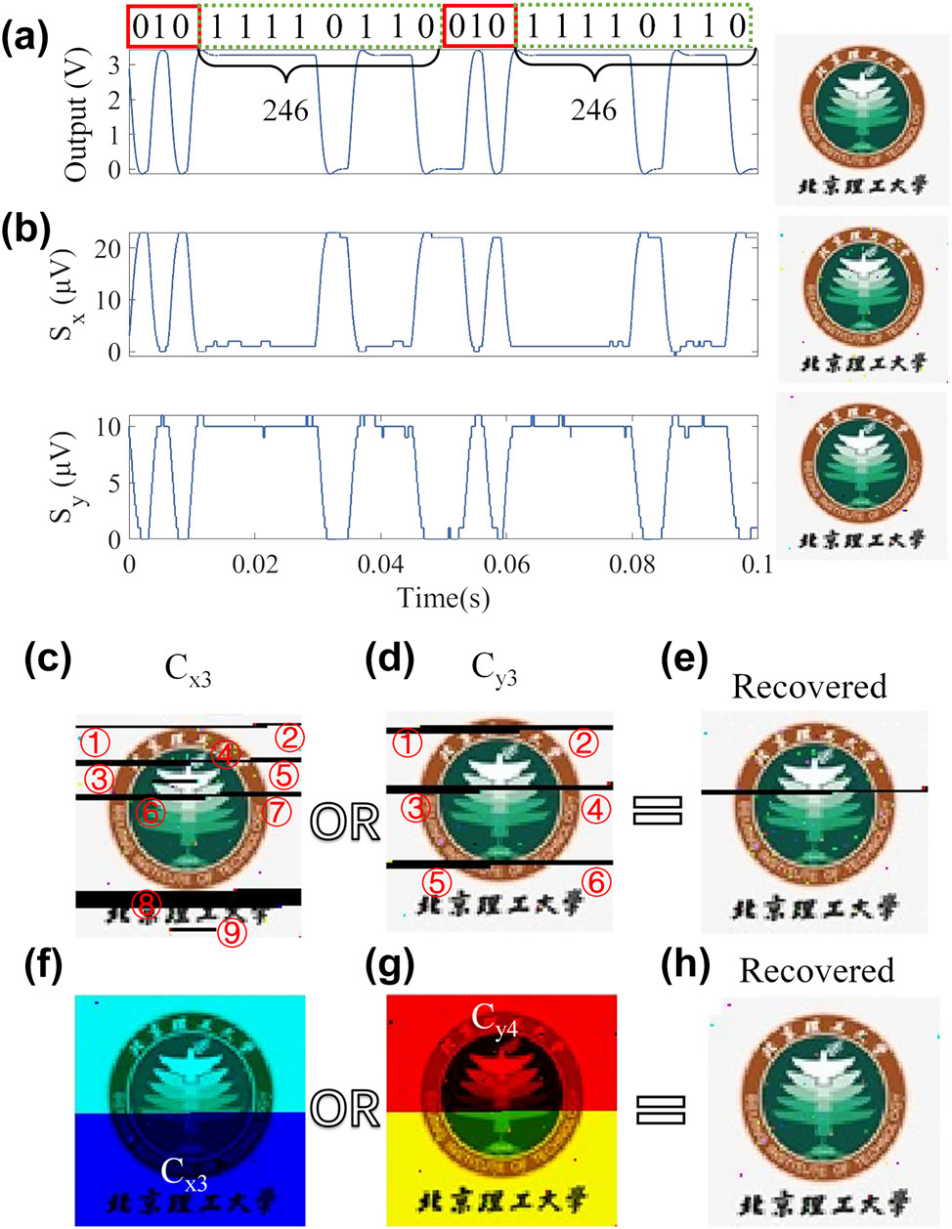
Communication measurement of the device. (a) Transmitted image data by Raspberry Pi. (b) Received image data in the *x*-polarization and *y*-polarization channels of CH_3_. The illustrations on the right are decoded images of different channels. (c)–(e) Realization of anti-interference in THz communication by spatial diversity through CH_3_. (f)–(h) Improvement of data capacity by spatial multiplexing of the *x*-polarization channel (CH_3_) and *y*-polarization channel (CH_4_).

We then assessed the performance of the PDM-MIMOS in THz communication by transmitting a 24-bit image with 80 × 80 pixels, as shown in the right inset of Figure 10a. Figure 10b depicts the detected binary signals of the red-blue-green image through channels C_y3_ and C_x3_. Meanwhile, Figure 10a shows the output electrical signal by Raspberry Pi after DAC. To make it possible to identify a valid signal after a communication interruption. We inserted the “010” identifier between 8-bit color data. The red box in Figure 10a is the identification code encoded at a frequency of 167 Hz, while the green dashed box is the color data encoded at 100 Hz. The encoding frequency of the identification code and the signal is different, which helps to distinguish the identification code and the signal after communication interference. Since the pins of Raspberry Pi can only output a maximum voltage of 3.3 V, the output electrical signal in Figure 10a was amplified 5 times by the amplifier and then applied to CH_3_ to achieve simultaneous modulation of C_y3_ and C_x3_ channels. S_y_ and S_x_ are the image data transmitted simultaneously through the C_y3_ and C_x3_ channels. The voltage level of the *x*-polarized THz signal was the opposite of the output signal and the *y*-polarized THz signal. The reason is that the chemical potential is reduced after the high gate voltage corresponding to the code “1” is applied to the graphene. Then, a high-intensity *y*-polarized wave and a low-intensity *x*-polarized wave are obtained.

After the communication transmission was completed without interference, the data were decoded to restore the images, as shown in the insets of Figure 10b. Only a few pixels show information loss. The BERs for C_y3_ and C_x3_ transmissions are 0.64 % and 1.8 %, respectively, which are significantly higher than the results shown in Figure 9. After checking the Raspberry Pi’s original output data, we noticed that the reason is the occasional loss of information when transferring data through the Raspberry PI GPIO data port.

Next, we assessed the polarization spatial diversity of the PDM-MIMOS. To simulate the situation of communication interruption during directional communication, we used a metal plate that randomly blocked C_y3_ and C_x3_ channels when transmitting image data. The C_x3_ channel was interfered by the blocking with nine times, and the C_y3_ channel was interfered with six times, resulting in severe distortion after being independently restored, as indicated by red numbers in Figure 10c and d. The interfered data were assigned the value of “0,” then the data from the two channels were performed logical OR to recover the correct information (e.g., 00011011 || 01000011 = 01011011). Therefore, image recovery can be achieved if one of the two channels has collected the correct data. By utilizing the spatial diversity, the data of dual channels were used to restore the image, and the results are shown in Figure 10e. Although some pixels cannot be restored due to the complete loss of information in both channels, most data are restored successfully. Next, we performed the polarization spatial multiplexing by C_x3_ and C_y4_. Half of the data were transmitted through channel C_x3_, and the other half through channel C_y4_, as shown in Figure 10f and g. Finally, the restored image is shown in Figure 10h.

The above results indicate that the PDM-MIMOS can achieve polarization spatial diversity and multiplexing. Specifically, spatial diversity can realize anti-interference communication, spatial multiplexing can double the communication transmission rate, and the dual channels communication of orthogonal polarization will not interfere with each other.

We also transmitted text information over the channels C_y1_ and C_x1_ of CH_1_, as shown in Figure S4 (see Supporting Information).

### 4.4 Reliability of communication

**Figure 11:**
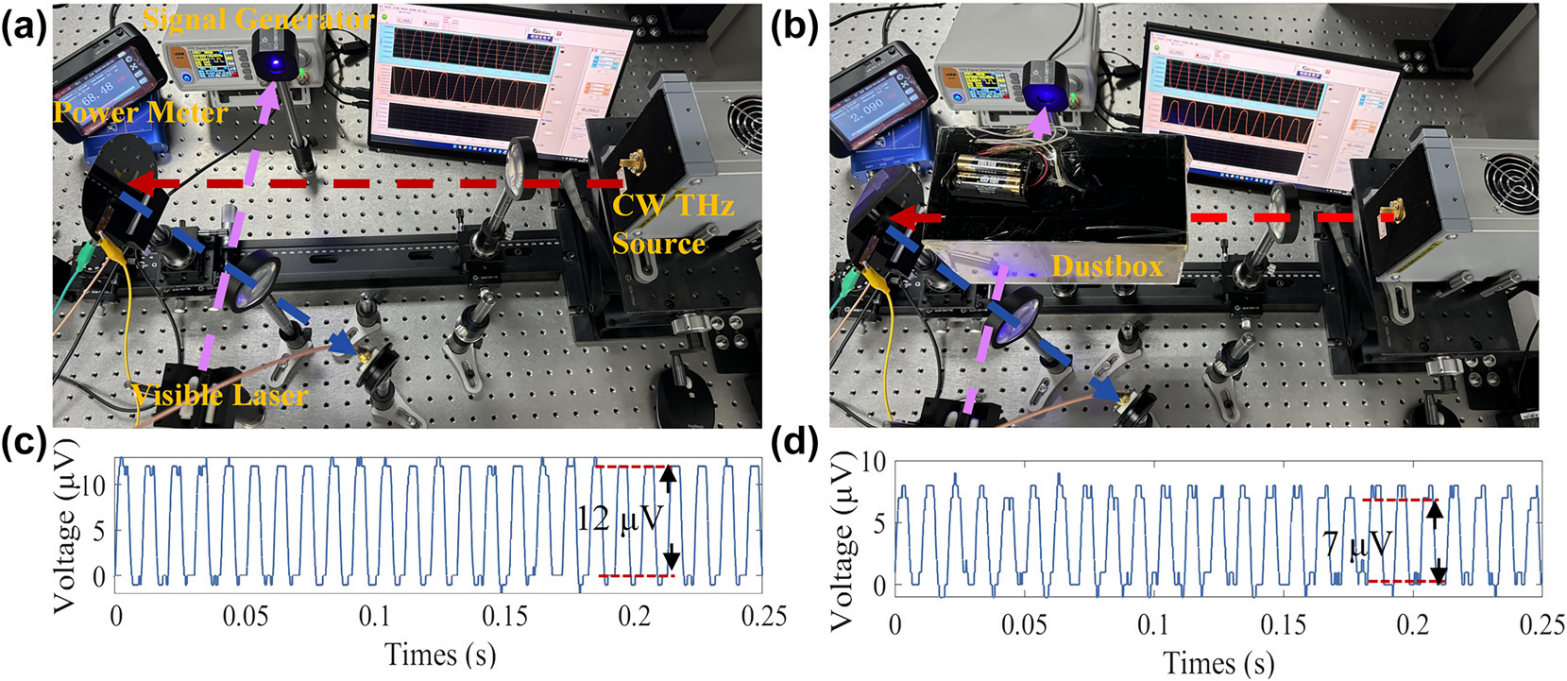
Signal attenuation comparison between visible light signal (*λ* = 400 nm) and THz signal in dust storm environment. (a)–(b) Signal transmission measurement in indoor and dust storm environments. The sandstorm environment is simulated by a dust box. *P*_*s*_ = 2.09 mW, *P*_*i*_ = 68.46 mW. (c) The transmission results of THz signal in the indoor environment and (d) dust storm environment.

Although Free Space Optical (FSO) communication systems also have a very wide available bandwidth at infrared and visible light frequencies, several issues limit the practicality of these solutions in wireless communication. For example, dust particles will affect the signal propagation [47]. After measuring polarization spatial diversity and multiplexing, we assessed the THz communication performance under a dust storm. To more intuitively demonstrate the impact of dust storms on visibility and optical communication, we used a 400 nm laser source and measured it with a power meter (GCI-08), as shown in Figure 11a. The measured laser power without the dust storm was *P*_*i*_ = 68.46 mW. Meanwhile, we loaded a square wave signal by the signal generator to modulate the THz wave over the C_x3_ channel, and the detected peak-to-peak value was 12 μV, as shown in Figure 11c. To simulate the dust storm, we made an acrylic display box of 20 cm × 10 cm × 10 cm with high transmittance. The box was sealed with dry dust (dimension range from 20 μm to 150 μm) and a fan that blows the dust up to mimic the conditions of a dust storm, as demonstrated in video 2 (see Supporting Information). Then, the dust box was added to the communication system, as shown in Figure 11b.

The measured power of the laser with the dust box was attenuated to *P*_*s*_ = 2.09 mW, and the peak-to-peak value of the THz signal was attenuated to 7 μV, as shown in Figure 11d. Because the power detected by the UFD is proportional to the output voltage, it can be calculated that the power of the visible light signal is reduced to 3 %. In comparison, the THz signal remains at 58.3 %, which is 19.4 times the remaining intensity of the visible light signal.

The results show that the anti-interference ability of THz communication in villainous weather is more robust than optical communication.

## 5 Device comparison

Table 1 compares the overall size between the proposed THz PDM-MIMOS and other THz MIMO devices.

**Table 1:** Comparison among different THz MIMO devices.

Ref.	MIMO antenna	*f* (THz)	Channel n umber	MIMO size (*λ*_0_^2^)	Separation	Beamforming	Experiment
[33]	Graphene	1	4–16	NA	0.1 mm (0.33*λ*)	Yes	No
[35]	Graphene	10–20	4	53.3 × 40	0.24 μm (8–16*λ*)	Yes	No
[48]	CMOS	0.14	4	0.78 × 0.82	1.1 mm (0.5*λ*)	No	Yes
[37]	Horn antennas	0.0876	9	58.4 × 58.4	10 cm (29.2*λ*)	No	Yes
[38]	Horn antennas	0.1	4	33 × 33	8 cm (26.7*λ*)	No	Yes
**This work**	Graphene metasurface	0.289	8	22.7 × 22.7	0–2 mm (1.93*λ*)	Yes	Yes

It is evident that traditional PDM-MIMOS THz communication systems require two transmit (TX) horn antennas to transmit dual-polarized THz waves, and each horn antenna can only modulate one polarization direction, which limits the flexibility and integration of the system [37], [38]. Additionally, the MIMO modulator based on CMOS offers the advantages of a small size and a high communication rate. However, due to the limitation of the processing accuracy of PCB boards (3 mil – 5 mil), it is challenging to achieve beamforming of THz waves [48]. Therefore, it is only commonly used for short-range wireless communication (<0.25 m), or combined with fibers to achieve communication [49], [50]. In contrast, MIMO devices based on graphene metasurfaces have significant advantages in terms of small size, high integration, and the ability to realize beamforming [33], [35]. However, due to fabrication and material limitations, graphene-based MIMO metasurfaces designed in previous works still face challenges in practical applications. To address this issue, the approach proposed in this work uses a single metasurface to simultaneously modulate dual-polarization of THz waves and perform beamforming in different directions, which not only overcomes the implementation difficulties of graphene-based devices but also effectively reduces the device size.

## 6 Conclusions

In summary, we propose a graphene-based polarization spatial diversity and multiplexing 2 × 2 MIMO modulator, which is experimentally proven can be used in the THz communication at 0.289 THz. The advantage of PDM-MIMOS is that the output and input THz signals with different polarization directions do not interfere with each other; thus, the distance between adjacent RX can be significantly reduced. Meanwhile, by introducing an F–P cavity, the converted intensity of the *y*-polarized wave is enhanced by 11–21 times. It is experimentally demonstrated that each metasurface can communicate through two channels with orthogonal polarization directions and different propagation directions. The data rate of communication is 120 Kbpbs, which is a low data rate compared to other works (>Gbps) [37], [38]. The reason for the low cut-off frequency is the large graphene area and the high resistance of the slightly doped silicon substrate. The use of top-gate modulated patterned graphene in future research holds great promise for significantly increasing the modulation speed (>1 GHz) [46], [51]. Also, we use the PDM-MIMOS to perform the anti-interference and multiplexing, which is suitable for the THz frequency band communication. Besides, under the dust storm environment, the reliability of THz communication is 19.4 times stronger than that of visible light communication. We anticipate that the proposed PDM-MIMOS THz modulator can provide an effective strategy to enhance the robustness and capacity of THz communication, which can be applied to THz wireless communication, detection, and imaging.

## Supplementary Material

Supplementary Material Details
