# Supplementary material for: Polarization spatial diversity and multiplexing MIMO surface enabled by graphene for terahertz communications
Source: Nanophotonics. 2025 Jul 15;14(17):2909–22. doi: 10.1515/nanoph-2025-0204 (PMC12397746; doi:10.1515/nanoph-2025-0204)
Supplement: Supplementary file 1 — Supplementary Material Details [file j_nanoph-2025-0204_suppl_001.docx]

Polarization Spatial Diversity and Multiplexing MIMO Surface Enabled by Graphene for Terahertz Communications

Jianzhou Huang^1,2^, Xudong Wu^1,2^, Chenjie Xiong^1,2^, Jia Zhang^1, 2,3^, and Bin Hu^1,2*^

1. School of Optics and Photonics, Beijing Institute of Technology, Beijing 100081, China.

2. National Key Laboratory on Near-surface Detection, Beijing, 100072, China.

3. China Academy of Aerospace Science and Innovation, Beijing 100089, China.

*Correspondence and requests for materials should be addressed to B. H (E-mail: [hubin@bit.edu.cn](mailto:hubin@bit.edu.cn))

**Table S1**: Selected Unit cells

| No. | *θ* (°) | *β* (°) | E_y_ Phase (π) |
| --- | --- | --- | --- |
| Superlattice 1 (N=8) | | | |
| 1 | 20 | 45 | 2.06 |
| 2 | 70 | 45 | 1.8 |
| 3 | 110 | 45 | 1.55 |
| 4 | 140 | 45 | 1.32 |
| 5 | 190 | 45 | 1.05 |
| 6 | 70 | -45 | 0.8 |
| 7 | 110 | -45 | 0.55 |
| 8 | 140 | -45 | 0.32 |
| Superlattice 2 (N=18) | | | |
| 1 | 20 | 45 | 2.06 |
| 2 | 40 | 45 | 1.95 |
| 3 | 60 | 45 | 1.85 |
| 4 | 80 | 45 | 1.74 |
| 5 | 100 | 45 | 1.63 |
| 6 | 120 | 45 | 1.46 |
| 7 | 140 | 45 | 1.32 |
| 8 | 160 | 45 | 1.21 |
| 9 | 180 | 45 | 1.1 |
| 10 | 20 | -45 | 1.06 |
| 11 | 40 | -45 | 0.95 |
| 12 | 60 | -45 | 0.85 |
| 13 | 80 | -45 | 0.74 |
| 14 | 100 | -45 | 0.63 |
| 15 | 120 | -45 | 0.46 |
| 16 | 140 | -45 | 0.32 |
| 17 | 160 | -45 | 0.21 |
| 18 | 180 | -45 | 0.1 |


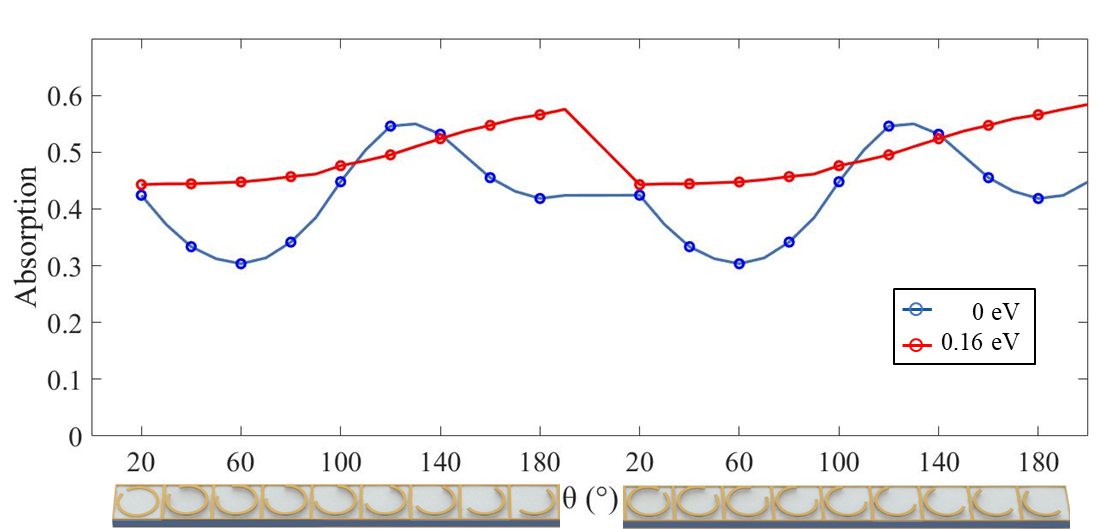


**Figure S1**: Absorption of different unit cell structures under different graphene chemical potentials (*f*=0.289 THz).


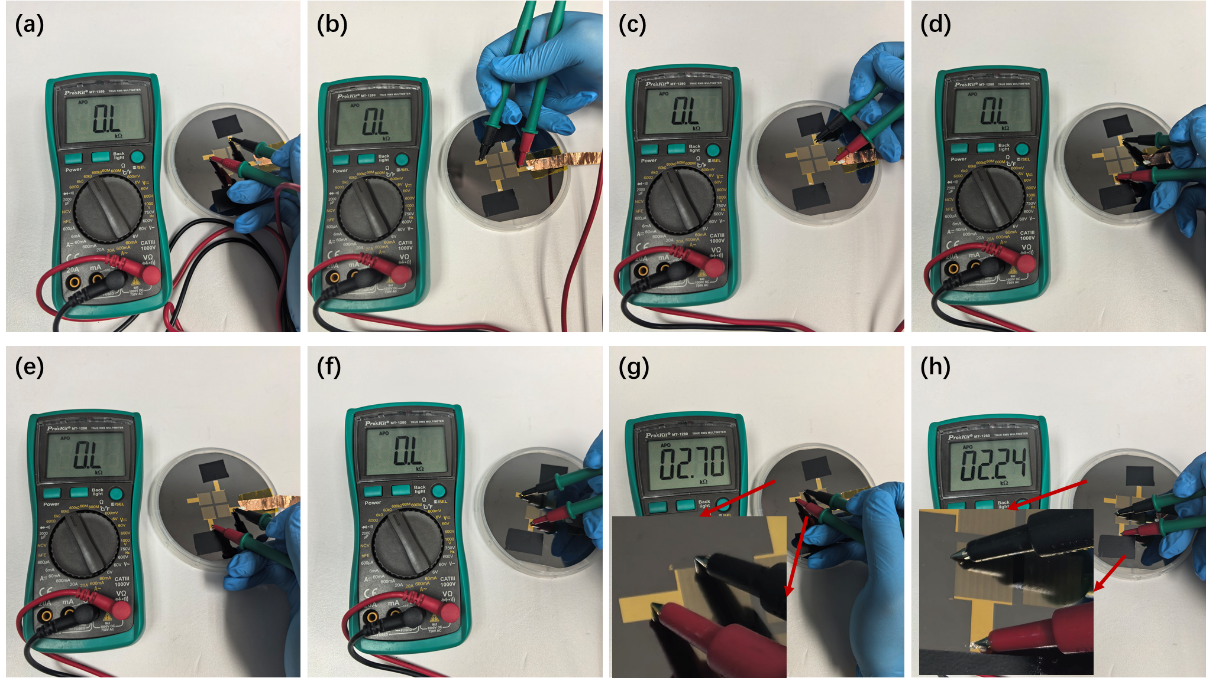


**Figure S2**: Electrical isolation measurement between different graphene regions. (a)-(f) Electrical isolation between graphene-covered metasurfaces CH_1_-CH_4_. (g)-(h) Conductivity between the electrode and graphene.

Electrical isolation between metasurface CH_1_-CH_4_ was measured as shown in Figure S2(a)-(f), confirming mutual isolation across all four metasurface pairs. This demonstrates that alcohol-swab wiping effectively achieves graphene segmentation and electrical isolation. Additionally, electrode-to-graphene conductivity measurements (Figure S2(g)-(h)) were performed using red and black probes contacting the electrode and off-metasurface graphene, respectively. The results indicate conductive behavior between the electrode and graphene, with resistance ranging from 2 to 3 kΩ.


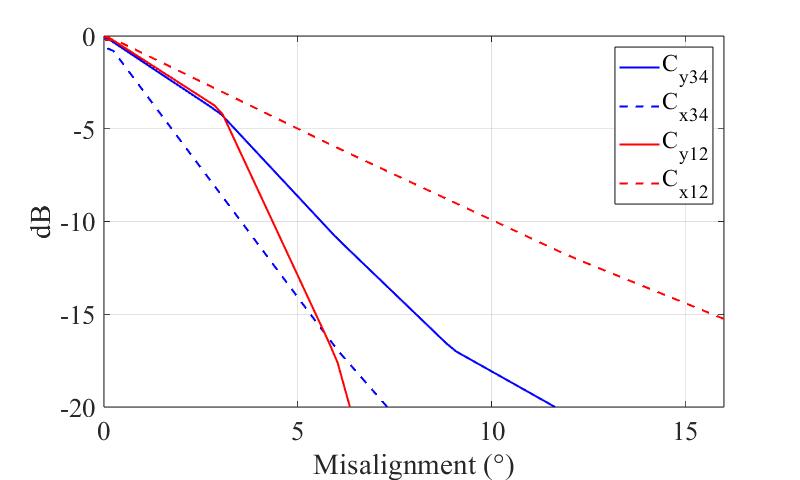


**Figure S3:** Sensitivity to misalignment. Calculated based on the data of radiation patterns.


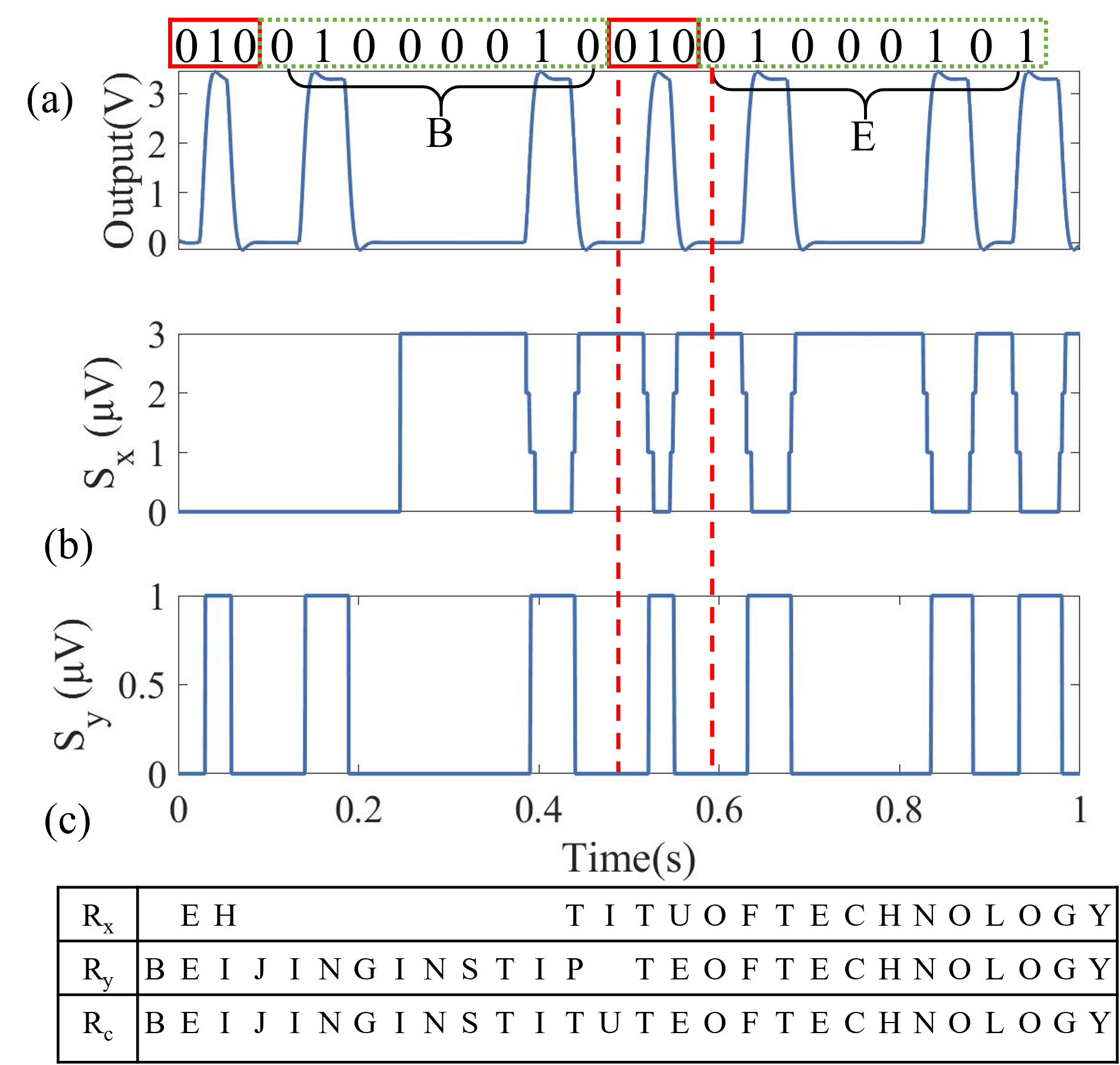


**Figure S4:** (a) Output ASCII code data by Raspberry Pi. (b) Received ASCII code data with interference. (c) Decoded results of the *x*-polarization (R_x_) and *y*-polarization (R_y_) channels, respectively. Rc is the

S_y_ and S_x_ are the text data transmitted simultaneously through the C_y1_ and C_x1_ channels. Figure S4(a) shows the output of Raspberry Pi. Same as in the above experiment, we used the metal plate that randomly blocks C_y1_ and C_x1_ channels when transmitting text data to simulate the situation of communication interruption during directional communication. As depicted in Figure S4(b), the correct information of 'B' was lost in the communication over the C_x1_ channel. Meanwhile, the identification code ‘010’ was also lost. In this case, the ASCII code ‘00000000’ was used to replace this whole 8-bit byte, which is the null character ‘NUL’ in ASCII code. Nevertheless, the correct data (01000010) in the C_y1_ channel can be used for text restoration. The correct ASCII code for "B" can be obtained by performing logical OR (00000000 || 01000010 = 01000010). Figure S4(c) demonstrates the restoration through the polarization spatial diversity. R_x_ and R_y_ are text transmitted separately under communication interference, resulting in incorrect restoration. However, the correct text information can be recovered by performing the logical operation of OR on the data transmitted through two channels, as depicted in R_c_.
